# HEALTH – ALIMENTATION – WELFARE

**Published:** 2011-08-25

**Authors:** F Popa

**Affiliations:** Carol Davila University of Medicine and Pharmacy Romania

The first international reunion based on the above-mentioned theme will take place between the 15th and the 17th of October, in Brasov, Romania. In fact, the event represents a continuation of ‘SANABUNA’ Conference which took place in Bucharest, in ‘Carol Davila’ University of Medicine and Pharmacy. The event approached the imperative of reflection and the responsible action towards the ‘health–alimentation–welfare’ problem, trying to underline that new and necessary direction of the attitudes is necessary, in order to permit the foundation of a public–private partnership, able to offer adequate solutions of influencing the behavioral changes to improve health and the economic–social responsibility. The pertinent interventions during the debates have shown the novelty of the partnership, having as a basis the good intention as well as the science in transforming the behavior that is able to produce aliments and permit choices of aliments in accordance with health and durability, understanding the interaction between the alimentary lifestyle and other lifestyle factors. 

The conclusion of the event was, that the speech and the action towards the ‘alimentation–health–welfare’ problem, must be modified in order to preserve our lives, by opening our minds and taking our spirits high, so as to cross ‘the ocean of distrust’, whose huge waves furiously strike the shores of the economic–social buildings, overcoming the fragments of understanding, interacting, getting involved, communicating and learning how to make the right change in our producers, distributors, individual consumers and organizational consumers behavior. [1] 

The Congress in Brasov will take place under the higher patronage of the Romanian Patriarchy and the Romanian Academy. Prestigious universities and institutions in Romania, in collaboration with The International Distribution Association –A.I.D.A. Bruxelles and EHI Retail Institute Koln, with the support of the Ministry of Public Health and the Ministry of Agriculture and Rural Development, will organize it.

Among the personalities from outside the country, who will honor this event, we must mention the following: Professor Eliot SOREL, M.D.,D.L.F.A.P.A., GWU School of Medicine and School of Public Health, Founder Conflict Management Section WPA, Washington, D.C., Professor Dr. Artemis Simopoulos, Department of Psychiatry and Behavioral Sciences, Stanford University School of Medicine, Professor Bernd Hallier Managing Director EHI Retail Institute in Köln, EHI trustee for GLOBALG.A.P, Member of the Consultative Council of ‘Transparent Food’, President Orgainvent, President of European Retail Academy ; Professor John L. Stanton Chair, Department of Food Marketing, Saint Joseph's University (the first endowed chair in food marketing in the USA) and founding editor of the Journal of Food Products Marketing, Philadelphia, Léon F. Wegnez, General Secretary of A.I.D.A. Bruxelles, Member of the French Academy of Commercial Studies and President of the Royal Belgian Distribution Committee. 

The interdisciplinary character of the manifestation will allow the covering of some aspects of certain novelty, starting from the harmonization of the preoccupations effectively connected to building a better life indeed, in the context of the pressure of realizing the connections between health, alimentation and the different aspects of business, and of the imperative of identifying the adequate answers in the confrontation with the welfare reform, reconfiguring consistent paths with fundamental values, the education being the core of adaptation, not being able to neglect the solidarity, amid the approach of both the activities and the actions which could improve the health and welfare, by building and sustaining the trust of the consumer in the alimentary chain, as well as in the manner of directing the brilliant minds in a durable relationship ‘Health–  Alimentation–Welfare’, making a current cultural preoccupation out of it, the aliments being a part of our culture. We are talking about a culture which: treats the health improvement together with the raise of the social responsibility; it stimulates the preoccupation for the synergy between the social backs and the business ones, raising reciprocity; recognizes the moment of truth and invites the architects of conversation to work together as a team, which generates a responsible action, by considering that there is a ‘therapist’ in each person who respects with responsibility the partnership for ‘alimentation–health–welfare’, a partnership which can bring back the trust in life and in the market. 

Because, we should not forget that the health of people is in the center of durable development and the adaptation of the business, consequently. 
